# Clinical and molecular characterization of early T-cell precursor acute lymphoblastic leukemia: Two cases report and literature review

**DOI:** 10.1097/MD.0000000000013856

**Published:** 2018-12-28

**Authors:** Xiao-Xue Wang, Danyang Wu, Lijun Zhang

**Affiliations:** Department of Hematology, The First Hospital, China Medical University, Shenyang, China.

**Keywords:** cytogenetics, ETP-ALL, molecular biology, prognosis, treatment

## Abstract

**Rationale:**

Early T-cell precursor acute lymphoblastic leukemia (ETP-ALL) is a small subtype of T-cell acute lymphoblastic leukemia with a typical immune-phenotype: lack of T-lineage cell surface markers CD1a and CD8 expression, weak or absent CD5 expression, at least one of the myeloid or hematopoietic stem cell markers. It is characterized by high rate of induction failure and the effective unified treatment strategies are still indeterminate. We present 2 ETP-ALL cases.

**Patient concerns:**

A 42-year-old man presented with abnormal hemogram for 4 months, intermittent fever for 2 months and cough for 1 week. A 27-year-old woman was admitted to the hospital for a fever and headache for that had persisted for 1 week.

**Diagnosis:**

The peripheral blood examination, the bone marrow aspiration and flow cytometry for both patients revealed ETP-ALL.

**Interventions:**

Both cases accepted chemotherapy including cytarabine.

**Outcomes:**

In case one, the patient reached complete hematological remission with negative minimal residual detected by flow cytometry after the first circle of chemotherapy. In case 2, the patient received complete remission after the second circle of chemotherapy with high doses of cytarabine.

**Lessons:**

The application of the high-dose cytarabine in induction chemotherapy of ETP-ALL can bring better outcome. ETP-ALL with myeloid features may benefit from therapies used in myeloid malignancies.

## 1 Introduction

Early T-cell precursor acute lymphoblastic leukemia (ETP-ALL) is a small subtype of early T-cell acute lymphoblastic leukemia (T-ALL) first proposed by Coustan-Smith et al^[1]^ in 2009 which may be originated from oncogenically transformed early T-cell precursors (ETPs), a subset of thymocytes representing recent immigrants from the bone marrow to the thymus which retain multilineage differentiation potential.

ETP-ALL accounts for about 11% to 15% of T-ALL with similar distributions among pediatric and adult cohorts and it is used to be reported to have poor response to chemotherapy, high frequency of induction failure or hematologic relapse, and inferior outcome.^[2]^ ETP-ALL presents with a unique immune-phenotype resembling to an ETPs: lack of T-lineage cell surface markers CD1a and CD8 expression, weak or absent CD5 expression, at least one of the myeloid or hematopoietic stem cell markers (CD13, CD33, CD34, CD117, CD11b, CD65, and human leukocyte antigen (HLA)-DR.^[3]^ Another feature of ETP-ALL is that it exhibits increased genomic instability and the genetic landscape is more closely to acute myeloid leukemia than lymphoid leukemia which suggests that it maybe responds poorly to lymphoid-cell directed therapy.^[4]^ Recently, several studies reported improved outcomes of ETP-ALL with the application of early intensified strategies, risk-directed treatment and allogenic hematopoietic stem cell transplantation^[5,6]^. Hereinafter, we are going to share our treatment experience of 2 ETP-ALL cases and summarize the progress of pathogenesis and treatment through literature review for ETP-ALL.

## 2 Case report

### 2.1 Case 1

**Figure 1:**
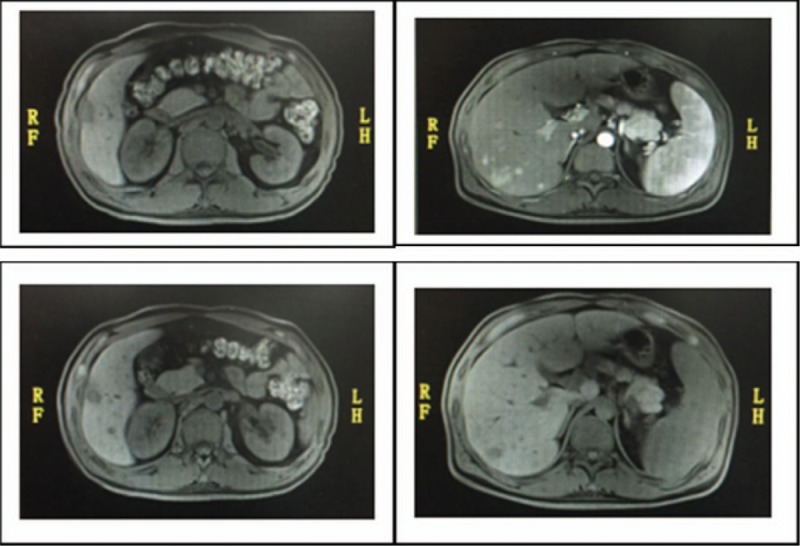
Multiple small round foci with long T1 and T2 signal intensity and annular post-contrast enhancement.

A 42-year-old man was admitted to The First Affiliated Hospital of China Medical University in February 2017 with abnormal hemogram for 4 months, intermittent fever for 2 months and cough for 1 week. The patient is an HBV carrier without family history of genetic or hematological disease. The peripheral blood examination showed a white blood cell count (WBC) of 25.99∗10^9/L, hemoglobin level (Hb) of 82 g/L and blood platelet count (PLT) of 103∗10^9/L. The liver and renal functions were normal. Routine ultrasound examination showed the patient with multiple lymphadenopathy involving cervical, supraclavicular, subclavian, axillary, inguinal, and posterior abdominal lymph nodes, in addition, multiple low-density foci was found on liver, the large one located on the inferior segment of the right posterior lobe about the size of 2.83∗2.84 cm. Further, the enhanced MRI showed multiple small round foci with long T1 and T2 signal intensity and annular post-contrast enhancement (Fig. 1).

The patient underwent BM aspiration which revealed 62.8% of nucleated cells were blasted. The blasts exhibited round, round-like or irregularly shape in different sizes, granular nuclear chromatin, 1 to 4 nucleoli and different amounts of cytoplasm, the large blasts with medullary morphology and the small ones showed lymphatic morphology. Typical Auer bodies also could be seen the some blasts. The features of cytochemical staining were 5% positive and 6% weakly positive for POX, positive for NAE and negative for NAF which can be seen in myeloid primitive cells and lymphatic primitive cells. PAS+ exhibited with diffuse tiny granules which are the feature of myeloid primitive cells rather than scattered thick granules in lymphocytes. In summary, the blasts presented both medullary features and lymphatic features (Fig. 2 ). Flow cytometry of the BM aspirate indicated that the blasts were presenting the stem cell markers CD34+, HLA-DR+, the T-cell markers cCD3dim+, CD2+, CD7+, CD5-, the B-cell markers CD19-,CD10-,and the myeloid cell markers CD13+, CD117+, CD14-, CD64-, CD33-, MPO-, suggesting a diagnosis of ETP-ALL. The chromosomal analysis of the BM cells was 46,XY,?t(5;12)(q33;p13)[10]/46,XY.^[10]^ The fusion genes listed in Table 1 were all negative. For gene mutation analysis, DNMT3A mutation and EZH2 mutation were detected in this case (Table 2).

**Figure 2:**
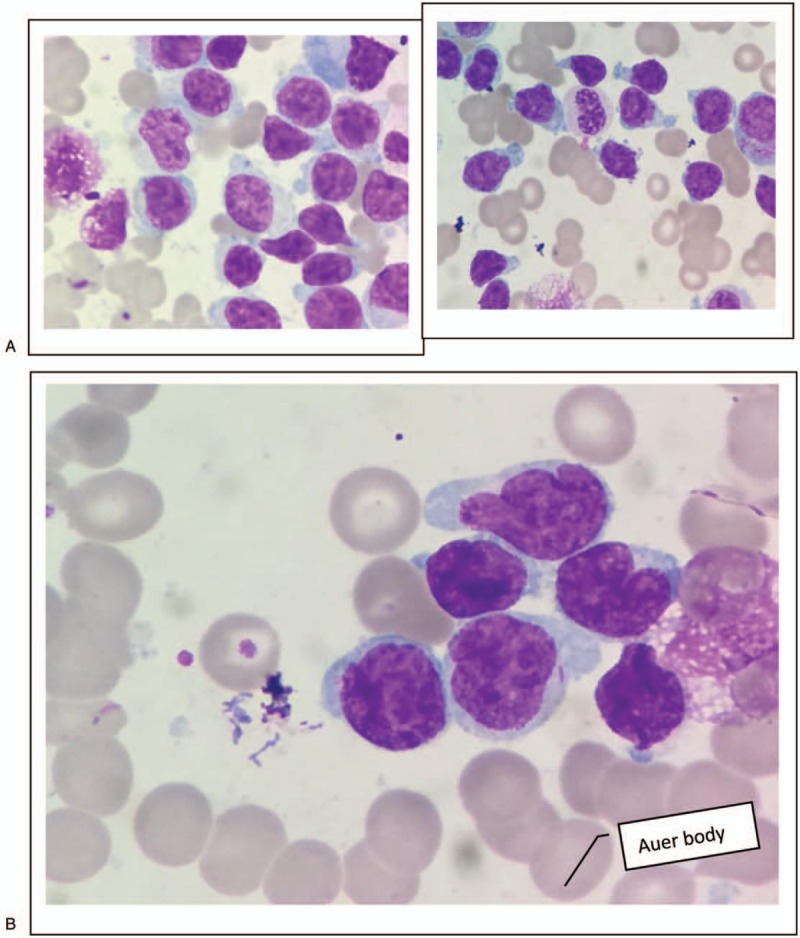
Cellular morphology of bone marrow in case 1. (A) The blasts exhibited round, round-like or irregularly shape in different sizes without typical characteristic, the large blasts with medullary morphology, and the small ones with lymphatic morphology. (B) Typical Auer bodies could be seen the some blasts. (C) The features of cytochemiscal staining was: 5% positive and 6% weakly positive for POX, positive for NAE and negative for NAF. PAS+ exhibited with diffuse tiny granules.

**Figure 2 (Continued):**
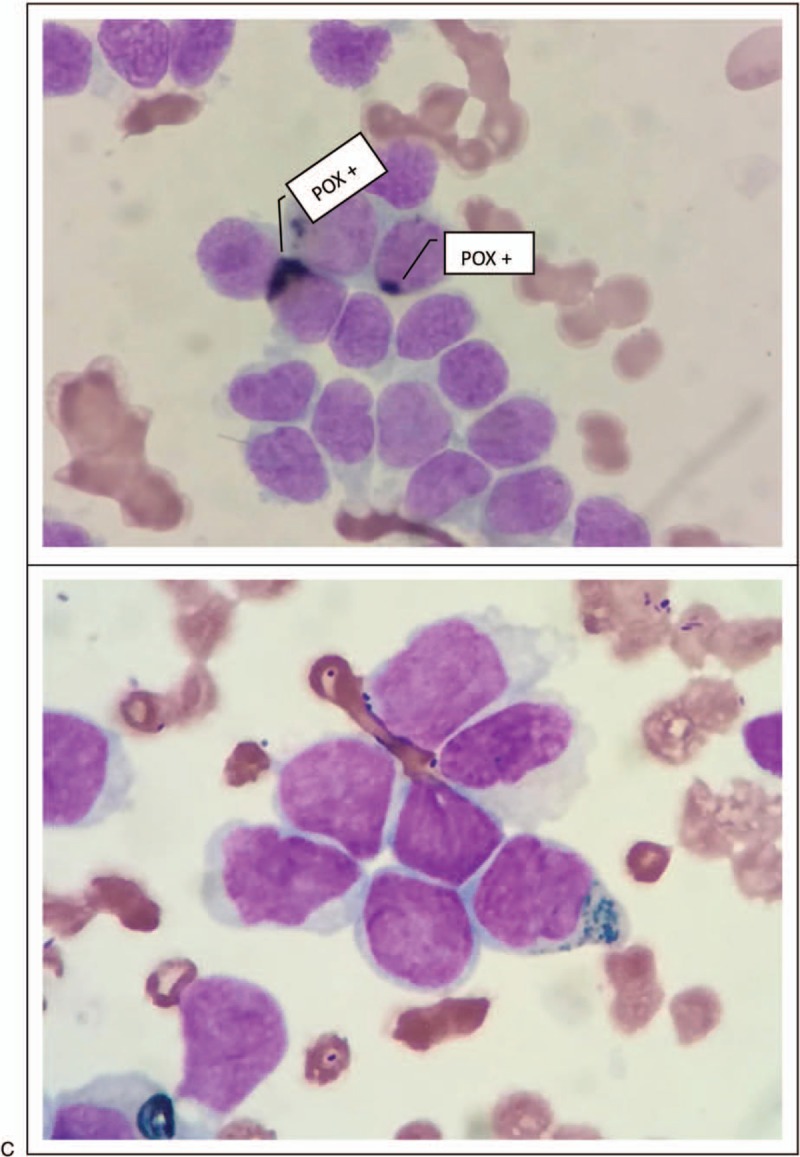
Cellular morphology of bone marrow in case 1. (A) The blasts exhibited round, round-like or irregularly shape in different sizes without typical characteristic, the large blasts with medullary morphology, and the small ones with lymphatic morphology. (B) Typical Auer bodies could be seen the some blasts. (C) The features of cytochemiscal staining was: 5% positive and 6% weakly positive for POX, positive for NAE and negative for NAF. PAS+ exhibited with diffuse tiny granules.

**Table 1:**
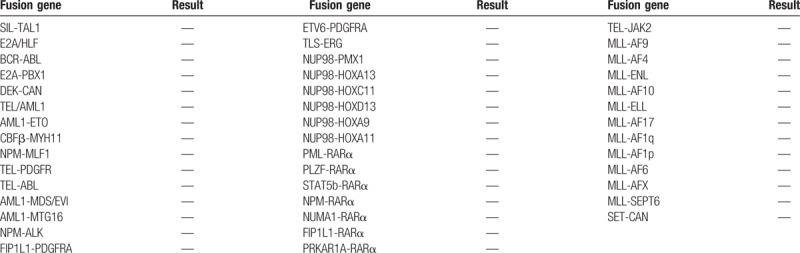
Fusion gene detection of case 1 and 2.

**Table 2:**
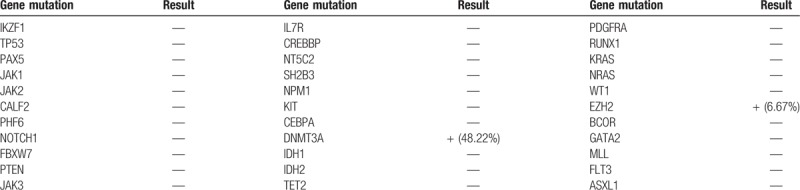
Gene mutation analysis of case 1.

**Figure 3:**
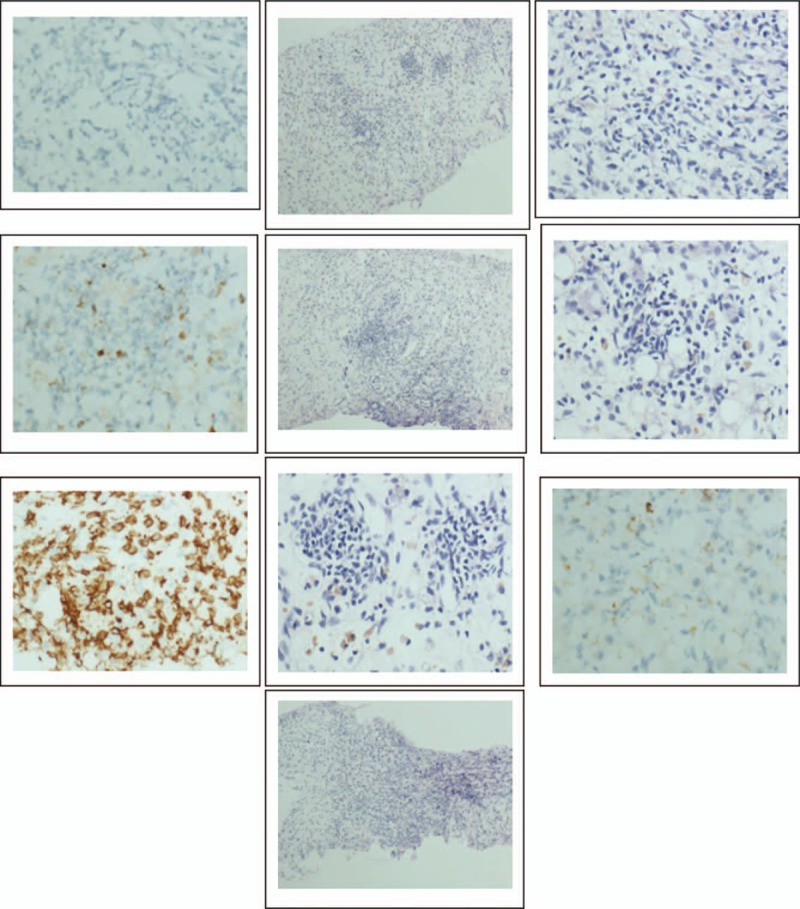
The immunohistochemistry of liver -occupying lesions: CK-, CD3+, CD20+, Pax-5(±), Bcl-2(+), CyclinD1(+), CD15(+), Ki-67(3%+), CD68(+), TdT(±), CD34(+) and CD117(+).

The patient received induction chemotherapy with vindesine, cytarabine, idarubicin, prednisone and pegaspargase (VADLP: 4 mg vindesine on days 1, 8, 15, 22; 180 mg cytarabine on days 4, 5, 6; 20 mg idarubicin on day 1 and 10 mg on days 2, 3, 15, 16; prednisone 100 mg on week 1, 80 mg on week 2, 60 mg on week 3 to 4; 5 mL pegaspargase on days 9 and 23). One month later, the routine blood examination was WBC 1.94∗10^9/L, neutrophils 0.59∗10^9/L, lymphocytes 1.29∗10^9/L, Hb 55 g/L and PLT 192∗10^9/l. BM aspiration revealed <5% lympho-blasts and no aberrant phenotypes were detected by flow cytometric immune-phenotyping which indicated that the patient had achieved a complete hematological remission with incomplete blood count recovery. Then the patient received a consolidation chemotherapy (VADLP: Consistent with the previous course of treatment). After 2 months, the routine blood examination of the patient was WBC 2.69∗10^9/L, neutrophils 1.09∗10^9/L, lymphocytes 1.28∗10^9/L, Hb 59 g/L and PLT21∗10^9/L, the BM aspiration and flow cytometric immune-phenotyping indicated the patient still with complete hematological remission. However the lesion in liver always existed without any change, considering the good condition of the patient, we arranged a needle biopsy for the liver-occupying lesions. The pathology exhibited fibrous tissue proliferation and heterotypic lymphocyte infiltration in which T-cells were predominant. And immunohistochemistry was presenting CK-, CD3+, CD20+, Pax-5(±), Bcl-2(+), CyclinD1(+), CD15(+), Ki-67(3%+), CD68(+), TdT(±), CD34(+), and CD117(+) (Fig. 3). The final date of follow-up was July 3, 2017, at which point the patient was alive and healthy.

### 2.2 Case 2

A 27-year-old woman in December 2016 took a visit to hospital who had a fever and headache for 1-week. The patient's peripheral blood was WBC 41.09∗10^9/L, Hb 72 g/L and PLT 83∗10^9/L. The proportion of blasts in peripheral blood was 32%. The liver and renal functions were normal and no abnormality was detected by abdominal ultrasound.

**Figure 4:**
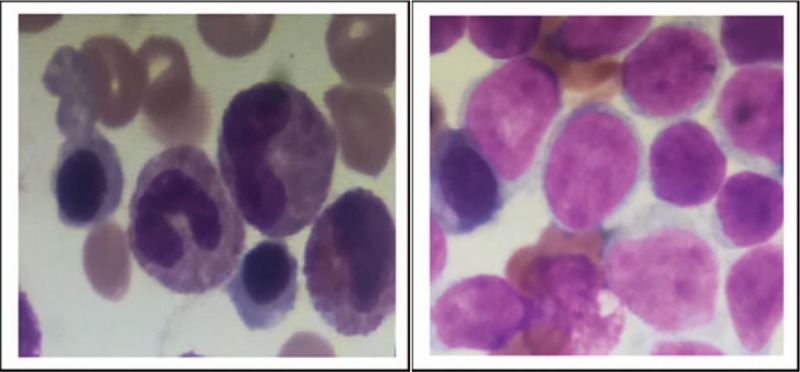
Histomorphological features of leukemic blasts from bone marrow aspirates. Blasts were round or round-like shape in different sizes (big cells in the majority), round or round-like nucleus, loose and granular nuclear chromatin, blurry nucleoli and different amounts of cytoplasm.

The patient underwent BM aspiration which revealed that the blasts proliferated actively accounted for 86% and exhibited round or round-like shape in different sizes (big cells in the majority), round or round-like nucleus, loose and granular nuclear chromatin, blurry nucleoli and different amounts of cytoplasm (Fig. 4). Cytochemical staining was weakly positive for POX, positive for NAE, positive for NAF, and 78% positive for PAS. Flow cytometry of the blasts were mainly CD33+, CD117+, CD7bri, partial CD34+, cCd3+, CD56+, CD38+, CD123+, but CD19-, CD10-, MPO-, CD5-, CD2-, CD13-, CD15-, HLA-DR-, CD1a-, CD64-, CD14-, CD3-, CD4-, CD8-, CD11c-, TdT-, suggesting a diagnosis of ETP-ALL. The fusion genes listed in Table 1 were all negative, for gene mutation analysis, NOTCH1 mutation and JAK3 mutation were detected in this case (Table 3). The chromosomal analysis was not performed.

**Table 3:**
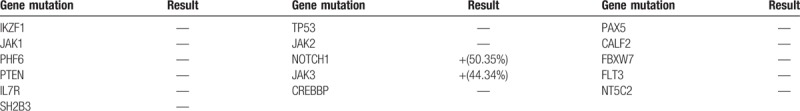
Gene mutation analysis in case 2.

The patient received induction chemotherapy with vindesine, daunorubicin, dexamethasone, cyclophosphamide, pegaspargase, (VCDLP: 4 mg vindesine on days 1, 8, 15, 22; 78 mg daunorubicin on days 1 to 3, 15 to 16; 1.2 g cyclophosphamide on day 1 and 15; 15 mg dexamethasone on days 1 to 12; 5 mL pegaspargase on day 8). One month later, routine blood examination were WBC 5.96∗10^9/L, neutrophils 5∗10^9/L, lymphocytes 0.56∗10^9/L, Hb 92 g/L, and PLT 413∗10^9/L. BM aspiration revealed 4% lympho-blasts and 3.01% aberrant phenotypes were detected by flow cytometric immune-phenotyping. Then the patient received the second course of chemotherapy (3000 mg cytarabine Q12 h on days 1–3; 10 mg dexamethasone on days 1–3; 5 mL pegaspargase on day 3; 4 mg vindesine on day 10; 4 g methotrexate on day 10). After 2 months, the routine blood examination of the patient was WBC 4.26∗10^9/L, neutrophils 2.5∗10^9/L, lymphocytes 1.02∗10^9/L, Hb 103 g/L, and PLT 369∗10^9/L. The BM aspiration and flow cytometric immune-phenotyping indicated the patient with complete hematological remission. The final date of follow-up was July 3, 2017, at which point the patient was alive and healthy.

## 3 Discussion and conclusion

ETP-ALL presented with typical immune-phenotype which is characterized by high rate of induction failure and without unified standard treatment options. Reviewing the literatures reported in the past few years, the studies mainly referred to 3 directions: prognosis of ETP-ALL versus typical T-ALL, genetic landscape of ETP-ALL and targeted therapy for ETP-ALL with specific mutations.

For prognosis of ETP-ALL versus typical T-ALL, different studies provided different conclusions listed in Table 4. Some findings suggest the ETP-ALL with obvious inferior outcome compared to the non-ETP-ALL, but several studies reported no difference in OS and EFS in these 2 cohorts.

**Table 4:**
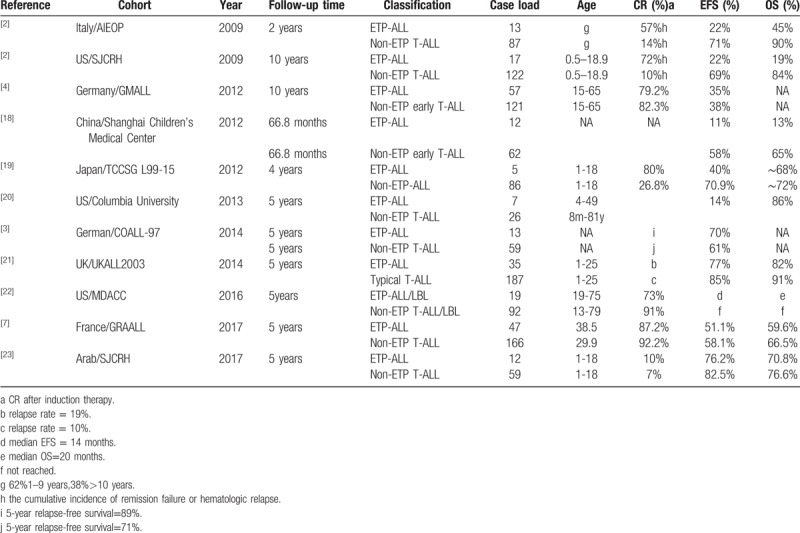
Survival data from published studies in ETP-ALL and non-ETP-ALL.

The genetic landscape of ETP-ALL was the research focuses in recent years. These studies indicated that the ETP-ALL showed increased genomic instability with myeloid features by performing gene expression profiling and mutational. Zhang et al^[7]^ performed whole-genome sequencing of 12 ETP-ALL cases and determined the recurrence and frequency of mutations in an extended 52 ETP T-ALL and 42 non-ETP T-ALL cases. They identified the most high frequency of mutations in genes regulating cytokine receptor and RAS signaling (67% of cases; NRAS, KRAS, FLT3, IL7R, JAK3, JAK1, SH2B3, and BRAF), in genes encoding key transcription factors involved in hematopoiesis (58%; GATA3, ETV6, RUNX1, IKZF1, and EP300) and in genes encoding histone modifiers (48%; EZH2, EED, SUZ12, SETD2, and EP300). Except the well-known genes involved in oncogenesis, some recurrent mutations including RELN, DNM2, and ECT2L were also identified. The same conclusion was obtained by Jonathan Bond et al^[8]^ that the rates of mutations in cytokine receptor and RAS signaling pathway genes (ETP, 62.2% v non-ETP, 37.8%; *P* = .008), mutations in hematopoietic development genes (ETP, 29.7% v non-ETP, 11.9%; *P* = .008) and mutations in histones modification (ETP, 48.6% v non-ETP, 29.6%; *P* = .03) is more higher in ETP-ALL than non-ETP. Besides a set of genes in DNA methylation (DNMT3A, IDH1, IDH2, TET2, TET3, and WT1) were also analyzed, but the rate of ETP-ALL was similar to non-ETP (ETP, 32.4% v non-ETP, 23.7%; *P* = .33).M Neumann et al^[6]^ analyzed the expression of 5 genes (BAALC, ERG, IGFBP7, WT1, and MN1) in ETP-ALL which are known to be with prognostic implications in T-ALL and AML compared with the remaining T-ALL. These 5 genes were all upregulated in ETP-ALL group which indicated the immature nature and poor outcome. For mutational analysis, they found NOTCH1 mutations which were the most frequent pathogenetic mutational event in T-ALL is rare and no FBXW7 mutations were found in ETP-ALL. In contrast, FLT3 mutations which show a low rate in the T-ALL were very frequently found in ETP-ALL. In another study Neumann M et al^[9]^ performed whole-exome sequencing in 5 adults with ETP-ALL, and analyzed the mutation status of selected genes (DNMT3A, EZH2, EP300, SH2B3, SUZ12) of 68 adult ETP-ALL patients in addition. Except ETV6, NOTCH1, JAK1, and NF1, they also identified some novel recurrent mutations (FAT1, FAT3, DNM2), and (MLL2, BMI1, DNMT3A) which are associated with epigenetic regulation. They also proposed adult ETP-ALL exhibits a different mutation spectrum from children, with a lower rate of PRC2 mutations and a higher rate of DNMT3A mutations.^[10]^

At present, there is no unified standard for the treatment options for ETP-ALL, most of which refer to the protocol for acute lymphoblastic leukemia, as specified in Table 5. Ribeiro et al^[11]^ verified more than 60% of adult patients with ETP-ALL harbor at least one mutation of DNMT3A, FLT3, or NOTCH1 which hint demethylating agents, kinase inhibitors, and g-secretase inhibitors maybe the new treatment for ETP-ALL. As in the study of Neumann et al^[12]^ T-ALL cell lines transfected with FLT3 expression constructs were particularly sensitive to tyrosine kinase inhibitors and they found ETP-ALL mutations with specific immunophenotype (CD2+, CD5-, CD13+, CD33-) and molecular pattern (aberrant expression of IGFBP7, WT1, GATA3, absence of NOTCH1 mutations and a low rate of clonal TCR rearrangements) which confirms the immaturity of ETP-ALL. As known more than 60% of ETP-ALL cases harbor gene mutations involved in kinase (JAK)/signal transducer and activator of transcription (STAT) signaling pathways, Maude et al.^[4]^ tested the effect of the JAK1/2 inhibitor ruxolitinib in xenograft models of ETP-ALL, showing that peripheral blast counts can be decreased independent of the presence of JAK/STAT pathway mutations or not, raising the therapeutic possibility of ruxolitinib in ETP-ALL. Lu et al^[13]^ tested the role of DNA methyltransferase inhibitor decitabine in pretreating ETP-ALL through the vitro test and suggesting that decitabine could enhance the chemosensitivity in ETP-ALL.^[14]^ Sachiko Kawashima-Goto^[15]^ suggested the Bcl2 inhibitor (ABT-737) can recover the sensitivity to prednisolone in leukemic cells with a high expression of MEF2C. Based on this, inhibition of Bcl2 might become a therapeutic candidate for ETP-ALL patients. NOTCH1 mutations occurred in a minority of ETP-ALL cases, Knoechel et al^[16]^ first apply γ-secretase inhibitors (GSIs) BMS-9060 to a relapsed refractory ETP-ALL patient and achieved complete hematologic response. Padi et al^[17]^ identified a high overexpression of PIM kinases in the majority of ETP-ALL, they applied PIM inhibitors to treat ETP-ALL through tumor xenograft experiments reaching an unsatisfactory outcome because PIM inhibitors can stimulate ERK and STAT5 phosphorylation. Then they found combining TKIs Ponatinib with PIM inhibitors can decrease the tumor burden of the mice which means a novel treatment strategy for ETP-ALL.

**Table 5:**
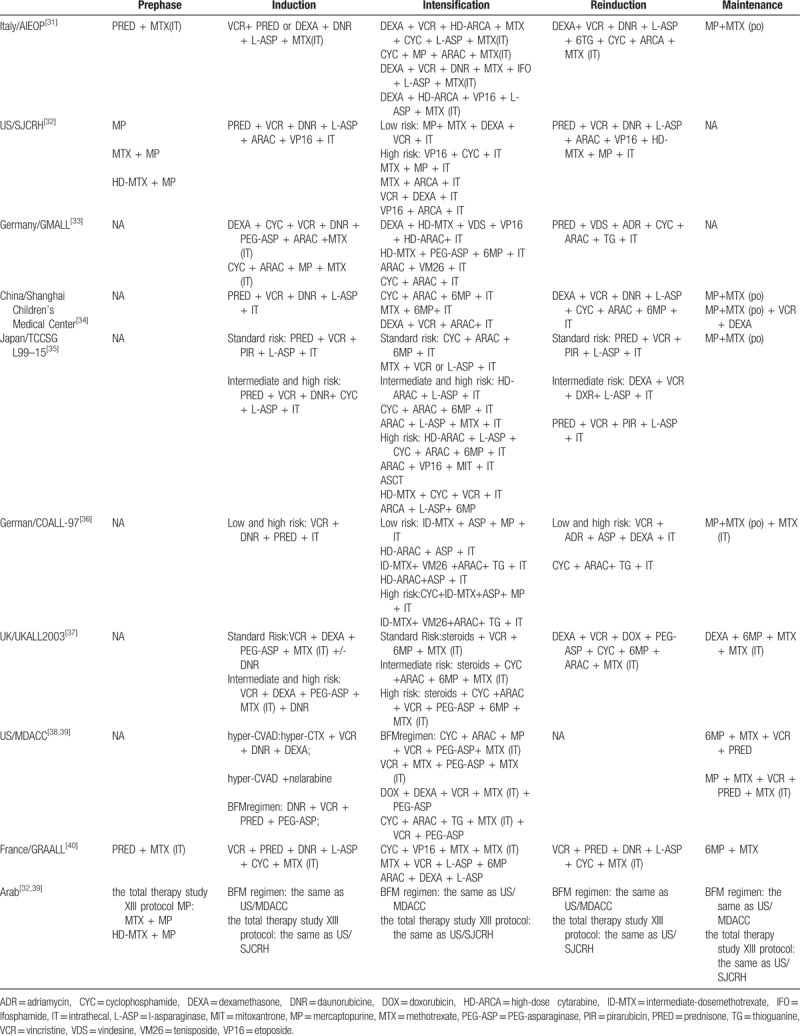
Treatment regimens used in the studies.

Since the specific immune-phenotype of ETP-ALL resembles to ETPs, it used to be thought ETP-ALL originates from ETPs. ETPs are a subset of thymocytes early immigrating from the BM to the thymus, except T-lymphoid potential they also keep myeloid cell differentiation potential.^[18]^ However, a mouse model study suggests a different scenario that ETP-ALL might arise from mature T-cells.^[19]^ Study conducted by Berquam-Vrieze et al^[20,21]^ also indicated ETP-ALL may originate from more differentiated cells than ETPs.

To clear the etiology of ETP-ALL except the cell originates the key events that initiate leukemia is also important. Although the genetic landscape of ETP-ALL has been identified but which one is the key process to drive malignant transformation is still unclear. Treanor et al^[22]^ proposed activating mutations in the interleukin-7 receptor are sufficient to generate ETP-ALL like disease in murine model which highly resembles human ETP-ALL. Goossens et al^[23]^ reported ZEB2 might be a leukemic driver to ETP-ALL which works through upregulating the interleukin-7 receptor expression and activation of the JAK/STAT pathway. Ezh2 inactivation results in enhanced expression of genes highly expressed in human ETP-ALL in the study of Danis et al^[24]^ MEF2C is associated with the response to glucocorticoid treatment, and the dysregulation of MEF2C in T-ALL leads to a similar gene signature to ETP-ALL.^[5]^ However, in the study of Colomer-Lahiguera et al^[25]^ MEF2C dysregulation is not necessarily to an ETP-like cell surface marker profile. They proposed the combination of CDKN1B deletions with the expression of MEF2C considered as a driving oncogene in ETP-ALL. And Kawashima-Goto et al^[15]^ suggested BCL2 inhibitor (ABT-737) may be a restorer of prednisolone sensitivity in ETP-ALL with high MEF2C expression. Lmo2 is an oncogenic transcription factor that is frequently over-expressed in T-ALL including ETP-ALL which must combine with its partner SCL or LYL1 to drive leukemia. Matthew et al^[26]^ identified ETP-ALL exhibited high expression of LYL1 but not SCL, and proposed LMO2 and LYL1 to be a driving factor of ETP-ALL, and inhibition of this interaction is an optional therapeutic approach.

The 2 cases of ETP-ALL in this report exhibited the similar immunophenotype and gene mutations with the other studies.^[3]^ Both of them showed sensitivity to cytarabine therapy, especially the first one, which involved both medullary and lymphatic systems in the induction phase. The application of the standard-dose cytarabine which is always used to treat AML brought an inspiring outcome: complete hematological remission with zero minimal residual disease (MRD). It is also consistent with the literature posted that ETP-ALL has the myeloid features and may benefit from therapies used in myeloid malignancies.^[9]^ Although the initial induction therapy of the second patient did not reach complete remission, the remission was achieved after moderate dose cytarabine reinduction therapy. These results suggest that cytarabine may have a good curative effect on ETP-ALL.

The OS and EFS of ETP-ALL compared to typical T-ALL in different studies listed above were conflicting. Several reasons may explain this phenomenon. One is the definition and classification of ETP-ALL were un-unified. For example, in UKALL2003 the ETP-ALL included the patients with CD5 positive or unknown, the number of definitive ETP-ALL was only 11.^[27]^ And in MDACC cohort the research subjects includes both ETP-ALL and ETP-LBL, besides there was no difference in outcomes between ETP-LBL and non ETP-LBL.^[3]^ The second reason has been mentioned above, it might be that our growing attention to the implementation of MRD based, risk-adapted treatment strategies can abrogate the poor prognosis of ETP-ALL. The last reason may be the genetic heterogeneity of ETP-ALL, for example, PRC2 mutation is an independent predictor of poor outcome.^[28]^ However, due to the short follow-up time and the lost visit in these 2 patients, it is not possible to observe EFS and OS well. More cases should be summarized in the future.

But anyway, the high rate of induction failure and MRD positive were exact, the development of an effective clinical management strategy is necessary. There are no common disease-specific mutations and gene expression profile in ETP-ALL, many studies listed above have researched some novel agents such as ABT-737, ruxolitinib, TKIs for targeted change of ETP-ALL and received a lot of inspiring outcomes. So next generation sequencing and whole genome sequencing to identify the multiple target genes of ETP-ALL is necessary for personalized treatment and MRD monitoring.^[29]^ A new technology of CAR T-cell therapies has a relatively good outcome for B-cell malignancies compared to T-cell.^[30]^ Since so many novel strategies are in research, more large-sample studies are needed to determine the efficacy of new drugs in the treatment of ETP-ALL. We believe that in the near future, the high resistance to treatment of ETP-ALL will be overcome.

## Author contributions

**Data curation:** Danyang Wu.

**Formal analysis:** Xiao-Xue Wang, Lijun Zhang.

**Resources:** Danyang Wu.

**Visualization:** Lijun Zhang.

**Writing – original draft:** Xiao-Xue Wang, Danyang Wu.

**Writing – review & editing:** Xiao-Xue Wang, Lijun Zhang.
